# Long scan depth optical coherence tomography on imaging accommodation: impact of enhanced axial resolution, signal-to-noise ratio and speed

**DOI:** 10.1186/s40662-018-0111-4

**Published:** 2018-07-09

**Authors:** Yilei Shao, Aizhu Tao, Hong Jiang, Meixiao Shen, Dexi Zhu, Fan Lu, Carol L. Karp, Yufeng Ye, Jianhua Wang

**Affiliations:** 1 Bascom Palmer Eye Institute, University of Miami, Miami, FL USA; 2 School of Ophthalmology and Optometry, Wenzhou Medical University, Wenzhou, Zhejiang China; 3 Hangzhou First People’s Hospital, Hangzhou, China; 4 Electrical and Computer Engineering, University of Miami, Miami, FL USA; 5 Bascom Palmer Eye Institute, University of Miami, Miller School of Medicine, 1638 NW 10th Avenue, McKnight Building - Room 202A, Miami, FL 33136 USA

**Keywords:** Optical coherence tomography, Axial resolution, Signal-to-noise ratio, Accommodation, Anterior segment

## Abstract

**Background:**

Spectral domain optical coherence tomography (SD-OCT) was a useful tool to study accommodation in human eye, but the maximum image depth is limited due to the decreased signal-to-noise ratio (SNR). In this study, improving optical resolutions, speeds and the SNR were achieved by custom built SD-OCT, and the evaluation of the impact of the improvement during accommodation was investigated.

**Methods:**

Three systems with different spectrometer designs, including two Charge Coupled Device (CCD) cameras and one Complementary Metal-Oxide-Semiconductor Transistor (CMOS) camera, were tested. We measured the point spread functions of a mirror at different positions to obtain the axial resolution and the SNR of three OCT systems powered with a light source with a 50 nm bandwidth, centered at a wavelength of 840 nm. Two normal subjects, aged 26 and 47, respectively, and one 75-year-old patient with an intraocular lens implanted were imaged.

**Results:**

The results indicated that spectrometers using cameras with 4096 camera pixels optimized the axial resolutions, due to the use of the full spectrum provided by the light source. The CCD camera system with 4096 pixels had the highest SNR and the best image quality. The system with the CMOS camera with 4096 pixels had the highest speed but had a compromised SNR compared to the CCD camera with 4096 pixels.

**Conclusions:**

Using these three OCT systems, we imaged the anterior segment of the human eye before and after accommodation, which showed similar results among the different systems. The system using the CMOS camera with an ultra-long scan depth, high resolution and high scan speed exhibited the best overall performance and therefore was recommended for imaging real-time accommodation.

## Background

In the human eye, accommodation is the ability to provide clear vision during near tasks by increasing refractive power. With presbyopia and cataracts, the ability of the accommodation reduces [1]. Research to understand the mechanism of accommodation and to recover accommodative ability has attracted great interest among ophthalmic and optometric researchers. The accommodation apparatus located in the ocular anterior segment is a key component that generates the refractive power to focus on close targets [2, 3]. Biometry of the anterior segment is therefore critical to understand the mechanism of accommodation and discover the effective restoration of accommodation. Several techniques are available for imaging the ocular anterior segment in vivo including Scheimpflug photography, ultrasound biomicroscopy (UBM), magnetic resonance imaging (MRI), Purkinje imaging and optical coherence tomography (OCT) [4–26]. There are advantages and disadvantages for each of these approaches. Ultrasound can be used with water baths that may distort or depress the anterior surface and change the biometric measurements [8]. Scheimpflug photography requires dilation, a non-physiologic condition that limits the use of this method for studying accommodation, and Scheimpflug photography results in low-resolution [4–6]. Compared with other methods, MRI is a non-optical imaging technique with high cost and low resolution. It is relatively time-consuming, making it difficult to obtain dynamic images [5].

OCT is a non-contact, non-invasive technology with high scan speeds and high axial resolution. The spectral domain OCT (SD-OCT) has the capability to image accommodation in both static and dynamic states [10–23, 25]. However, the maximum image depth is limited due to the decreased signal-to-noise ratio (SNR) in SD-OCT, which prevents the wide use of SD-OCT with long scan depths. The ideal SD-OCT requires a good SNR through the entire scan depth and a good imaging resolution for the entire axial range of the anterior segment. The whole anterior segment image, which includes the cornea, the anterior chamber and the crystalline lens, is essential for optical correction of the images and automatic surface registration/detection to obtain biometric measurements. The dual channel approach and image switching were used to extend scan depth [16, 20, 27]. Recently, we reported a method to improve the SNR by overlapping two images acquired with an ultra-long scan depth SD-OCT with two alternative reference arm lengths for imaging the entire anterior segment in vivo [20, 25]. Using this method, the range of scan depth with normalized SNR reached more than 11 mm, which was enough to image the axial range of the entire anterior segment. Our previous approach with the spectrometer using a Charge Coupled Device (CCD) camera with 2048 camera pixels had a trade-off because only a portion of the full spectrum provided by the light source was used in trading the scan depth [20, 25]. In addition, the scan speed of our previous study was slow due to the speed limitation of the CCD camera used. As demonstrated in the literature, the latest Complementary Metal-Oxide-Semiconductor Transistor (CMOS) technology attained faster imaging speeds compared with the CCD technology. However, CMOS may be subject to lower sensitivity and higher noise [28]. Before further improvement on spectrometer designs can be materialized for imaging the entire anterior segment, the impact of axial resolution, SNR and speed with different spectrometer designs need to be better understood. The goal of this present work was to demonstrate the impact of these spectrometer designs on image qualities in the anterior segment biometry during accommodation.

## Methods

### OCT systems and performance

**Fig. 1:**
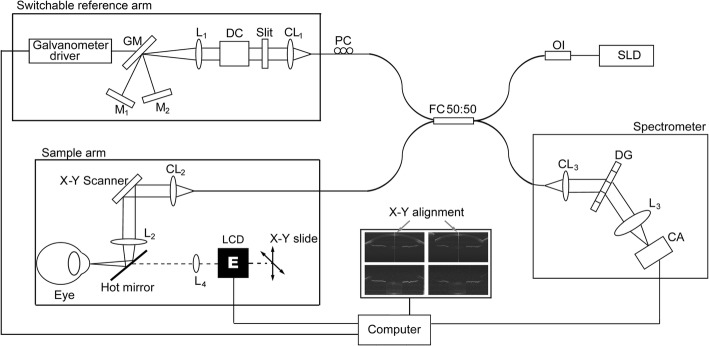
A schematic diagram depicting the spectral-domain OCT systems. SLD: superluminescent diode, OI: isolator, FC: fiber coupler, PC: polarization controller, CL_1–3_: collimating lenses, DC: dispersion compensator, L_1–4_: objective lenses, M_1–2_: refractive mirror, GM: galvanometer mirror, LCD: liquid-crystal display, DG: diffraction grating, CA: camera (CCD with 2048 pixels for system 1, CCD with 4096 pixels for system 2 and CMOS with 4096 pixels for system 3)

We tested three systems with different spectrometer designs including two CCD cameras and one CMOS camera. These three systems were based on the Michelson interferometer, which consists of a light source, a reference arm, a sample arm and a spectrometer, as diagrammed in Fig. 1. A superluminescent diode (SLD, InPhenix, IPSDD0808, Livermore, CA, USA) centered at a wavelength of 840 nm with a full-width at half maximum bandwidths of 50 nm was used as the light source. The power of incident light on the corneal surface of the human eye was 1.25 mW, which was well below the safe ANSI Z136.1 cut-off value. The beam was split into the sample arm and the reference arm using a 50:50 fiber coupler.

The three systems had a similar spectrometer design composed of four parts: a collimating lens (f = 50 mm, OZ Optics, Ottawa, Canada), a 1800 lines/mm volume holography transmission grating, an image enlargement lens with a focal length of 240 mm (f = 240 mm, Schneider Optics, Hauppauge, NY), and a line array camera. The three spectrometers were based on cameras with different data transfer rates and scan speeds (Table 1). The acquired interference spectrum data were transferred using the image acquisition board (PCI-1428 for system 1 and PCIe-1429 for systems 2 and 3, National Instruments, Austin, TX). A computer from Hewlett-Packard with an 8 GB RAM memory, an Intel Core 2 Quad processor and a Windows 7 64-bit operation system was used for the control and data acquisition of the OCT instruments. All OCT data acquisition drivers were developed in Labview (Version 2011, National Instruments, Austin, TX).

**Table 1:** Comparison of the different cameras used in the three optical coherence tomography systems

	System 1	System 2	System 3
Camera	CCD	CCD	CMOS
Mode	Aviiva-SM2010; E2V Technologies, NY, USA	Aviiva-SM2-CL-4010; E2V Technologies, NY, USA	Basler Sprint spL4096-140 k; Basler AG, Germany
Pixel size	10 μm	10 μm	10 μm
Number of pixels per A-line	2048 pixels	4096 pixels	4096 pixels
Maximum data acquisition rate	24,000 A-lines/s	12,000 A-lines/s	70,000 A-lines/s

**Fig. 2:**
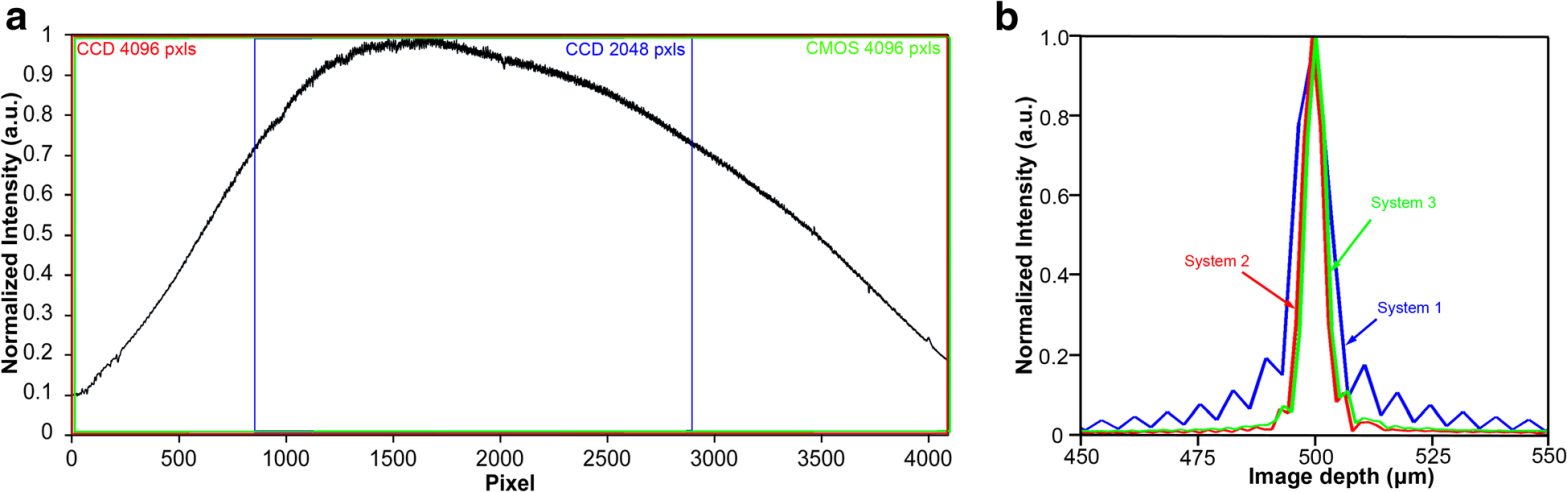
Spectrum of the light source captured by the three different systems (**a**) and the point spread functions (PSF) obtained using the three systems at a path difference of 0.5 mm (**b**). **a**: The areas of the available pixels from the cameras are indicated in blue (CCD with 2048 pixels), red (CCD with 4096 pixels) and green (CMOS with 4096 pixels) rectangles, respectively. **b**: Blue, the PSF of system 1 with the measured resolution of 10.9 μm in air; Red, the PSF of system 2 with the measured resolution of 7.0 μm in air; Green, of system 3 with the measured resolution of 7.0 μm in air

Figure 2a illustrates the spectrum of the light source captured by the three OCT systems. The calculated spectral resolution was 0.015 nm, which corresponds to a detectable scan depth of 11.76 mm in the air. The system performance including the real axial resolution and sensitivity were characterized by imaging a mirror in the sample arm at different positions. A neutral density filter with an optical density (OD) of 2.0 reduced the signal intensity. As mentioned elsewhere [12, 29], the resolution is indicated by the bandwidth of the point spread function (PSF). The signal intensity is represented with Fourier transformation in a logarithmic scale and sensitivity was calculated from SNR as

$$ sensitivity=10\times \log \left(\frac{S}{\sigma}\right)+20\times OD $$

where S is the signal peak, σ is the noise, and OD is 2.0 in this study.

**Fig. 3:**
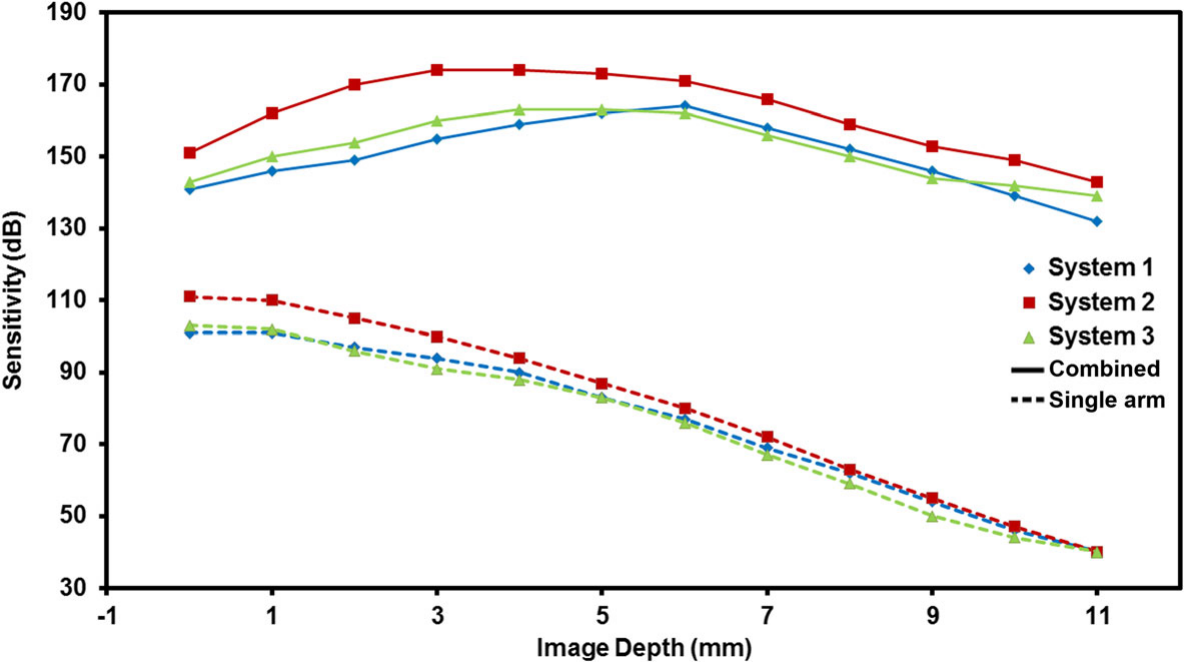
The sensitivity of the three systems measured at different image depths from the zero-delay line. Blue line, system 1 with CCD 2048 pixels; red line, system 2 with CCD 4096 pixels; green line, system 3 with CMOS. The solid line was the combined sensitivity acquired from two reference arms; the dotted line was obtained from a single arm

System 1 was based on our previously designed spectrometer and measured a scan depth of 12.34 mm. The scan speed was up to 24,000 A-scans per second, which was limited by the CCD line scan camera (2048 pixels; pixel size 10 μm; Aviiva-SM2010; E2V Technologies, NY, USA). The axial resolution was approximately 10.4 μm in air (Fig. 2b, blue line). The maximum sensitivity was 101 dB near the zero delay line with a 61 dB sensitivity drop at 11 mm (Fig. 3, blue line).

System 2 used a spectrometer based on a CCD camera with 4096 pixels per A-line (pixel size 10 μm; Aviiva-SM2-CL-4010; E2V Technologies, Elmsford, NY). The scan depth was 11.94 mm and the scan speed was 12,000 A-lines/s. Measured axial resolution was approximately 7.0 μm near the zero-delay line in air (Fig. 2b, red line). The sensitivity of the spectrometer was 111 dB near the zero delay line and had a 71 dB sensitivity drop at 11 mm (Fig. 3, red line).

System 3 used a spectrometer with a scan depth of 11.98 mm based on a CMOS camera that had a high scan speed of up to 70,000 A-lines/s (Basler Sprint spL4096-140 k; pixel size 10 μm; Basler Inc., Exton, PA). The axial resolution of the system near the zero-delay line was approximately 7.0 μm in air (Fig. 2b, green line). The sensitivity was 103 dB near the zero delay line and had a 63 dB sensitivity drop at 11 mm (Fig. 3, green line).

A special switchable reference arm was designed to acquire two images sequentially, similar to our previous study [20, 25] and others [16]. In this experiment, image overlapping was used for maximizing the SNR for the full image depth. This approach facilitates automatic registration and automatic boundary detection, which are currently under development. A galvanometer turned the light between the two mirrors mounted on the linear stages (M_1_ and M_2_ in Fig. 1) and was controlled by a square wave signal from the computer. Alterations between the two reference arms were synchronized with the scanning. The optical path difference (OPD) between the two arms determined the axial offset between the two frames, which was about 11 mm. The OPD was slightly adjusted with a linear stage so that the zero-delay lines of the two frames were placed on the top and bottom of the anterior segment for each individual [20, 25].

The sample arm was mounted on a modified slit-lamp microscope and used to adjust the image acquisition. An x-y galvanometer pair imaged the ocular anterior segment at the horizontal and the vertical meridians for alignment and acquisition using the custom acquisition software. To precisely align the scanning position, an X-Y cross aiming mode with 4 windows was used for live viewing. Two windows were used for viewing the images of the cornea and crystalline lens on the horizontal meridian and another two for viewing them on the vertical meridian. The operator monitored and adjusted the scanning position on both meridians in real time. Four images were acquired when the specular reflection was noted on both meridians, which ensured that the beam passed through the corneal apex. We used the cross-hair alignment live view to align the iris image on both horizontal and vertical scans so that the OCT beam was perpendicular to the iris plane (Fig. 1, insert). There is an angle between the visual axis and geometric axis of the eye known as the Kappa angle [30]. The OCT beam was aligned with the pupillary axis rather than the visual axis in the present study. In real-time, four images were quickly acquired, processed and displayed (Fig. 1). This real-time function avoided eye tilt and provided a better alignment of the eye during scanning. The focal plane of the beam was set at the anterior part of the crystalline lens by making on-axial adjustments of the objective lens (L_2_ in Fig. 1).

A liquid-crystal display (LCD) screen displaying a white Snellen letter “E” on a black background was set 10 cm from the tested eye. The target was controlled by a computer that altered the boundaries between a blurred or sharp picture. A trail lens (L_4_ in Fig. 1) in front of the LCD screen corrected for refractive error. The LCD and trail lens were combined and adjusted by a translation stage with a dual axis to make vertical and horizontal target adjustments.

### Experimental procedure and image analysis

This protocol was approved by the institutional review board for human research at the University of Miami Informed consent was obtained from each subject, and all patients were treated in accordance with the tenets of the Declaration of Helsinki. An eye from a 47-year-old male subject was first imaged using system 3 to test the instrument with the switchable reference arm.

The exposure time of the CMOS camera was set to 77 μs, which corresponds to a scan rate of 10,000 A-scans/s. The measurement lasted approximately 200 ms per frame to acquire a single image consisting of 2048 A-scans. The subject sat in front of the slit-lamp and looked forward at the internal fixation target “E” with near equivalent spherical refractive correction. After adjusting fixation to ensure the existing of the corneal apex both in the horizontal and vertical meridian for perfect alignment, a 14 mm cross-sectional scan was obtained.

**Fig. 4:**
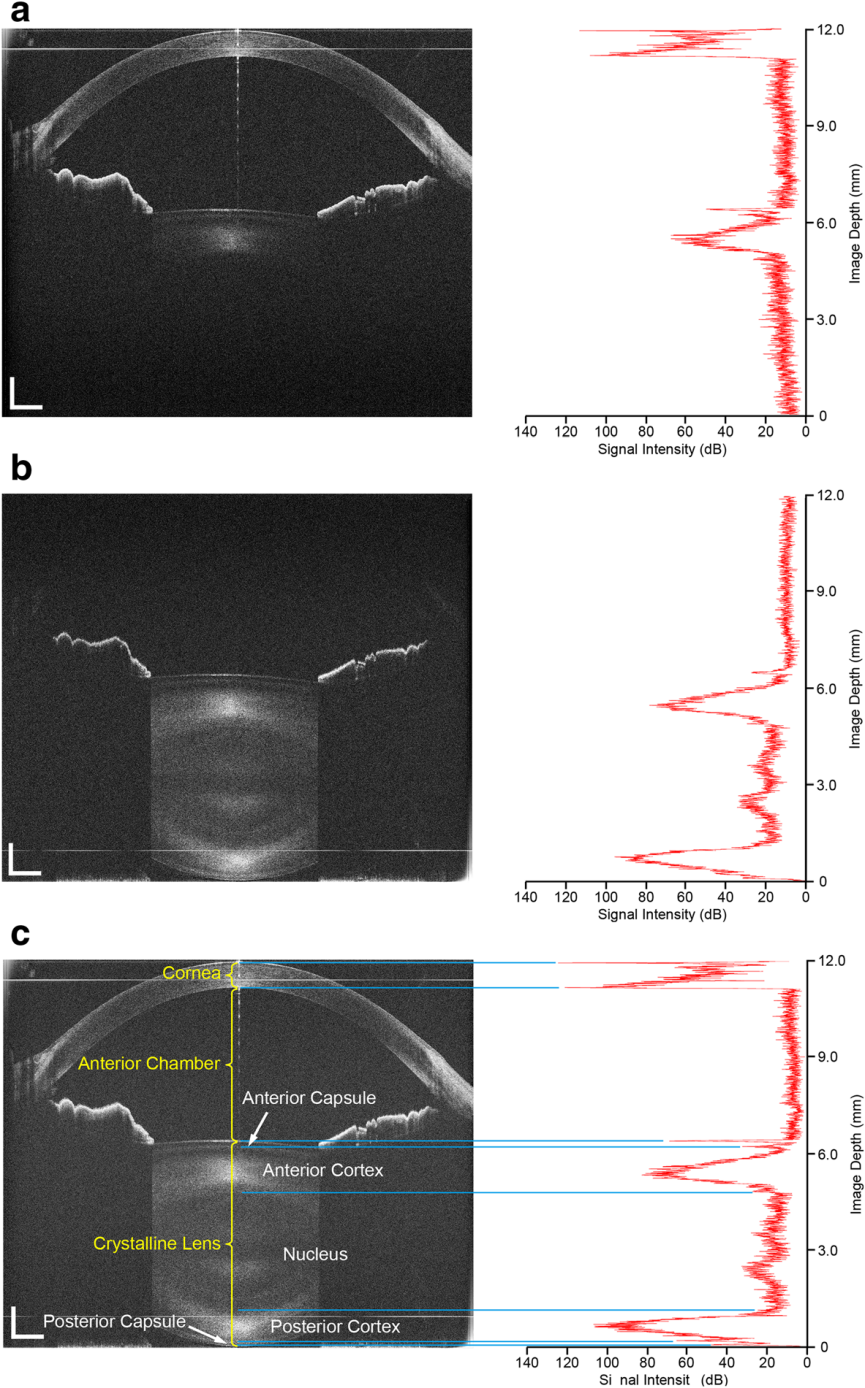
The images of the entire anterior segment from a 47-year-old subject was obtained and processed. **a**: The image and the longitudinal reflectivity profiles obtained from reference arm 1; **b**: The image and the longitudinal reflectivity profiles obtained from reference arm 2; **c**: The combined image obtained from overlapping image **a** and **b**, and the longitudinal reflectivity profiles through the whole anterior segment. Bar = 1 mm

Figures 4a and b show two single frames obtained from a 47-year-old subject using system 3 under relaxed conditions. The zero-delay planes were set at the top (Fig. 4a) and bottom (Fig. 4b) of the images, and showed the cornea, iris and the anterior part of the crystalline lens. There were also dim images of the posterior (a) and the entire lens without the cornea (b) because the signal-to-noise ratio decreased as showed in Fig. 3. The two frames clearly showed the common portion of the iris and the anterior surface of the lens and were then manually overlapped with the registration of common features using imaging software (Adobe Photoshop CS, Vision 8.0, Adobe Systems Inc., San Jose, CA). The common portion including the iris and the anterior surface of the crystalline lens was used for registration and overlapping the two frames. The rotation and translation between the two frames were adjusted and corrected during overlapping. In the overlaid image, the entire anterior segment including the anterior and posterior surfaces of the crystalline lens was clearly visualized, as well as the cornea, anterior chamber and iris (Fig. 4c). In this study, we selected the method of image overlapping but did not crop the part of the image with low sensitivity as described elsewhere [16]. This approach was beneficial for image registration because the human eye may have slight movement during image acquisition, and the rotation/translation between the two images could be realized with image registration. The offset between the two zero-delay lines was set at approximately 11 mm. Therefore the low SNR part of one arm was compensated by the high SNR part of another arm. The drop-off of the sensitivity was compensated through the entire scan depth as demonstrated in Fig. 3. In the combined image, the drop-off was calculated as the difference between the highest (at one of the position near the zero-delay line) and lowest (at the middle of the scan depth) sensitivities. The drop-off of the combined system was 21 dB (system 1), 28 dB (system 2) and 24 dB (system 3).

A custom-developed software produced the longitudinal reflectivity profiles during the first step of image analyses. Specular reflex on the corneal apex induces vertical hyper-reflective lines, interfering with image analysis [31]. The central 50 axial scans (approximately 0.36 mm width) were removed to avoid distortion of the central specular hyper-reflective reflex. The profiles of the 50 axial scans on either side of the anterior segment were also processed. The boundaries of the cornea and the lens were identified using the reflectivity profiles' peaks (Fig. 4c). The internal structure was identified by visualizing the cross-sectional images (Fig. 4c) for the purpose of demonstration. The central corneal thickness (CCT), anterior chamber depth (ACD) and central lens thickness (CLT) were also measured. Next, the boundaries of the cornea and the lens were outlined semi-manually using software specifically designed to construct the image. The custom-developed algorithm was used for each boundary correction and the refractive index of each medium (the refractive index of 1.387 for the cornea [32], 1.342 for the aqueous humor [33] and 1.408 for the crystalline lens [34] at 840 nm wavelength) was applied in this algorithm. Then, the curvature radii of the anterior and posterior surfaces of the cornea and lens were calculated. The algorithm for optical correction was validated in our previous study [25].

The three systems acquired the full range of the anterior segment in the left eye of a 26-year-old male subject. The refractive error in the tested eye was − 7.00DS/− 0.5 DC × 180. The images were obtained at both the horizontal and vertical meridian under relaxed and 4.00D accommodative states in a normal examination room and under dim light. The 2-dimensional cross sectional scans (B-scans) consisted of 2048 line scans (A-scans), using 2048 points per A-scan in system 1 or 4096 points in systems 2 and 3. To compare the three systems, the exposure time of each system was set at 4 times the initial value, which were 144 μs (systems 1 and 2) and 44 μs (system 3), which corresponds to the scan speeds of 6000 A-lines/s and 17,500 A-lines/s, respectively. It took approximately 333 ms per frame using systems 1 and 2, and approximately 114 ms using system 3.

The same subjects, a 26-year-old healthy subject and a 75-year-old patient with a monofocal intraocular lens (IOL, AcrySof SA60, Alcon) implanted, were dynamically imaged using the system 3 with the CMOS camera. In this case, the anterior segment length from the anterior surface of the cornea to the posterior surface of the IOL in the implanted patient was shorter than the phakic eye because the IOL was thin. Therefore, the distance between the two reference mirrors was decreased to place the zero-delay line of arm 2 near the posterior polar of the IOL. Thirty-one combined images with 1024 A-lines were continuously acquired for 3.72 s, with a single frame of 0.12 s and a frame rate of 8.3 frames per second. The OCT speed was 17,500 A-scan per second. The X-Y alignment was used but only horizontal images were obtained. The refractive correction during near vision was added to the trail lens. The target letter “E” was blurred at first to fog the eye and to relax the accommodation. The accommodative stimulus of 4.00D was set 1 s after scanning by altering the target from blurred to sharp. After outlining the peak intensity of the axial profile, as described above, the central corneal and crystalline lens/IOL thickness and anterior chamber depth were measured, and the results between the phakic eye and the IOL implanted eye were compared.

## Results

**Fig. 5:**
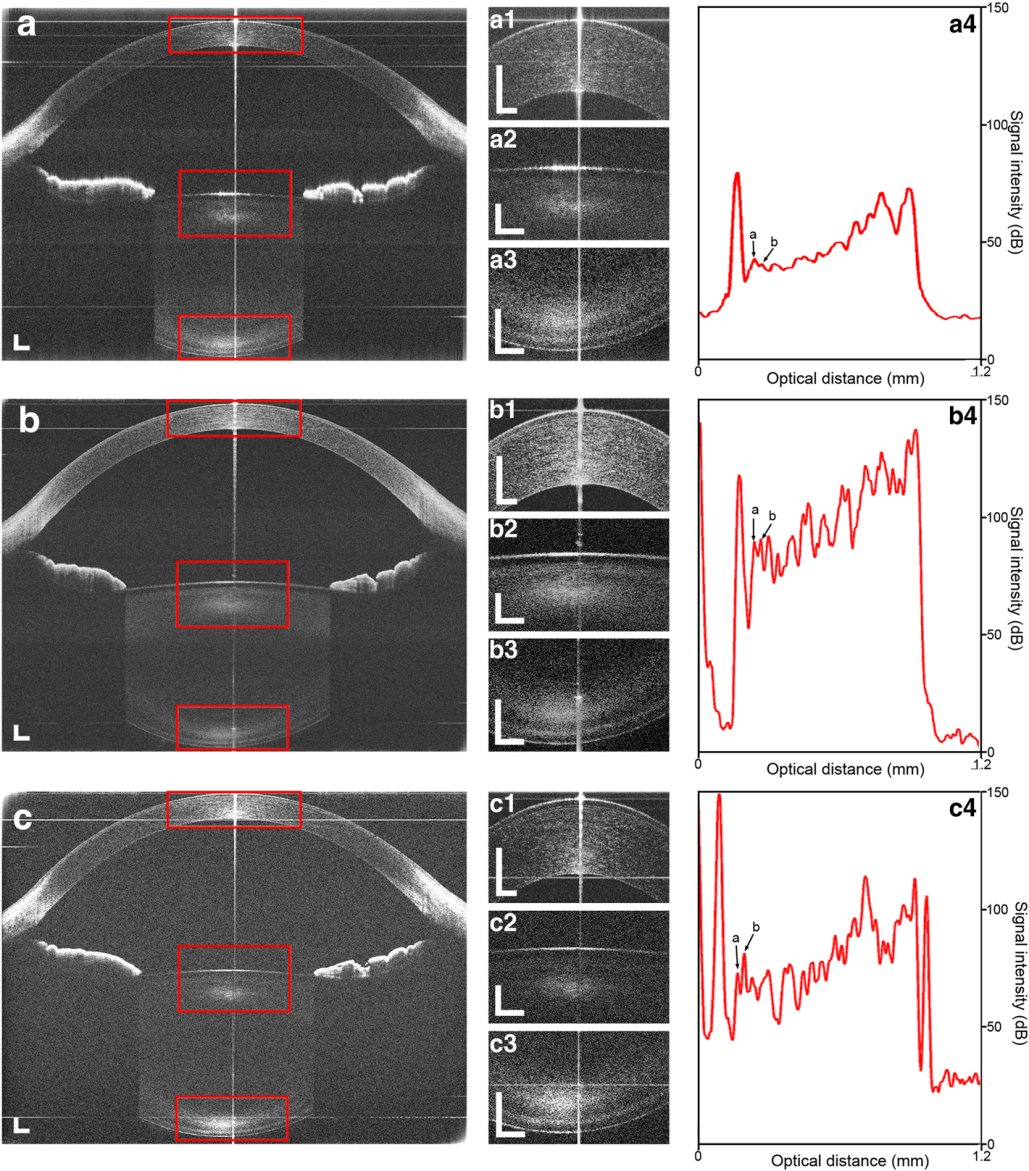
The uncorrected images taken from the entire anterior segment of a 26-year-old subject using the three systems. **a**: Image obtained by system 1 using a CCD camera with 2048 pixels; **b**: Image obtained by system 2 using a CCD camera with 4096 pixels; **c**: Image obtained by system 3 using a CMOS camera. a1-a3, b1-b3, c1-c3: The magnified images of the corneal apex (1), the anterior (2) and the posterior (3) of the lens surface using the three systems, respectively. a4, b4, c4: Longitudinal reflectivity profiles through the cornea. The boundaries of the Bowman’s layer were identified as the peaks a and b. Bar = 500 μm

**Fig. 6:**
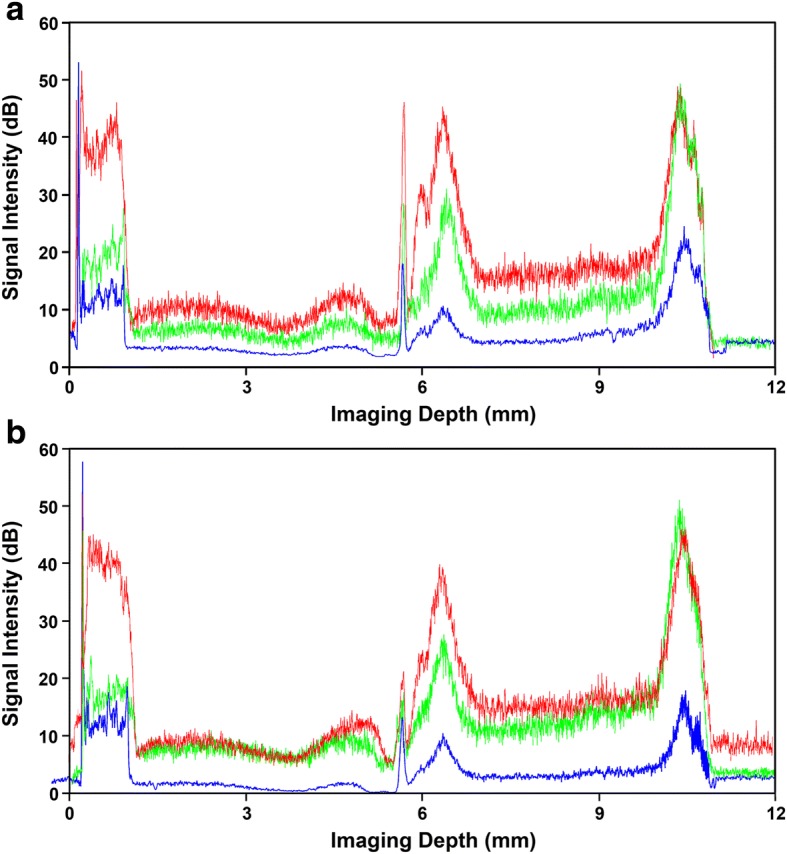
The longitudinal reflectivity profiles from a 26-year-old subject under the relaxed (**a**) and the accommodative (**b**) states. Blue line: Longitudinal profile obtained from system 1; Red line: Longitudinal profile obtained from system 2; Green line: Longitudinal profile obtained from system 3. The contrast scales were adjusted before obtaining the reflectivity profiles to demonstrate the peak locations representing the measured boundaries

Figure 5 depicts the combined OCT images from the left eye of the young subject with different systems. The image from system 2 using a CCD with 4096 pixels (Fig. 5b) resulted in the best contrast among the three devices due to its high sensitivity. Even though the background noise in the CMOS image appeared higher than that with the other instruments, the contrast was almost equivalent to that obtained with system 2 (Fig. 5c). The central Bowman’s layer in the magnified images was presented in systems 2 and 3 (Fig. 5b1 and c1), whereas the boundary of the corneal components in the image from system 1 was blurred (Fig. 5a1). Moreover, the boundaries of the Bowman’s layer in system 1 was barely identified as the peaks in the reflectivity profiles but was easily distinguished in systems 2 and 3 (Fig. 5a4-a4, peak a and b) [35]. The entire anterior segment was successfully visualized using both systems and the boundaries of the cornea and lens were clearly distinguished. Not only were the axial lengths across the full-length ocular anterior segment but the radii of the curvature of the cornea and lens were similar among these three OCT systems (Fig. 6 and Table 2).

**Table 2:** Anterior segment biometry obtained by the three devices under relaxed and accommodative states on the horizontal and the vertical meridian

		Relaxed state			Accommodative state		
		System 1	System 2	System 3	System 1	System 2	System 3
Horizontal meridian	CCT	0.55	0.55	0.55	0.55	0.55	0.55
	ACD	3.33	3.33	3.38	3.26	3.27	3.29
	CLT	4.77	4.73	4.76	4.81	4.80	4.80
	RAC	7.15	7.67	7.47	7.71	7.89	7.64
	RPC	6.29	6.72	6.46	6.54	6.86	6.63
	RAL	10.64	10.53	10.20	9.02	9.50	9.50
	RPL	5.88	6.16	6.12	5.33	6.14	5.67
Vertical meridian	CCT	0.54	0.54	0.55	0.55	0.54	0.55
	ACD	3.34	3.34	3.38	3.26	3.26	3.30
	CLT	4.78	4.73	4.77	4.82	4.79	4.81
	RAC	7.50	7.53	7.42	7.29	7.14	7.63
	RPC	6.21	6.30	6.13	6.14	6.04	6.27
	RAL	10.89	10.32	10.09	9.05	9.11	9.31
	RPL	6.39	6.32	6.56	5.98	6.30	5.70

Measurement in millimeters

*CCT* = central corneal thickness; *ACD* = anterior chamber depth; *CLT* = central lens thickness; *RAC* = curvature radius of the anterior corneal surface; *RPC* = curvature radius of the posterior corneal surface; *RAL* = curvature radius of the anterior surface of the lens; *RPL* = curvature of the posterior surface of the lens

**Fig. 7:**
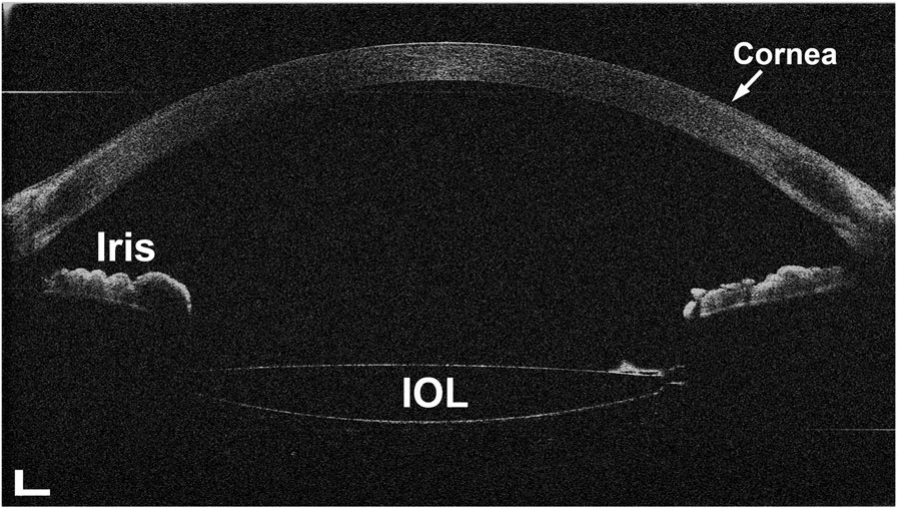
The uncorrected image of the anterior segment presented from a 75-year-old IOL implanted eye. The cornea, anterior chamber, iris and the IOL are clearly presented. The image consists of 1024 A-lines of 4096 pixels each. Bar = 500 μm

**Fig. 8:**
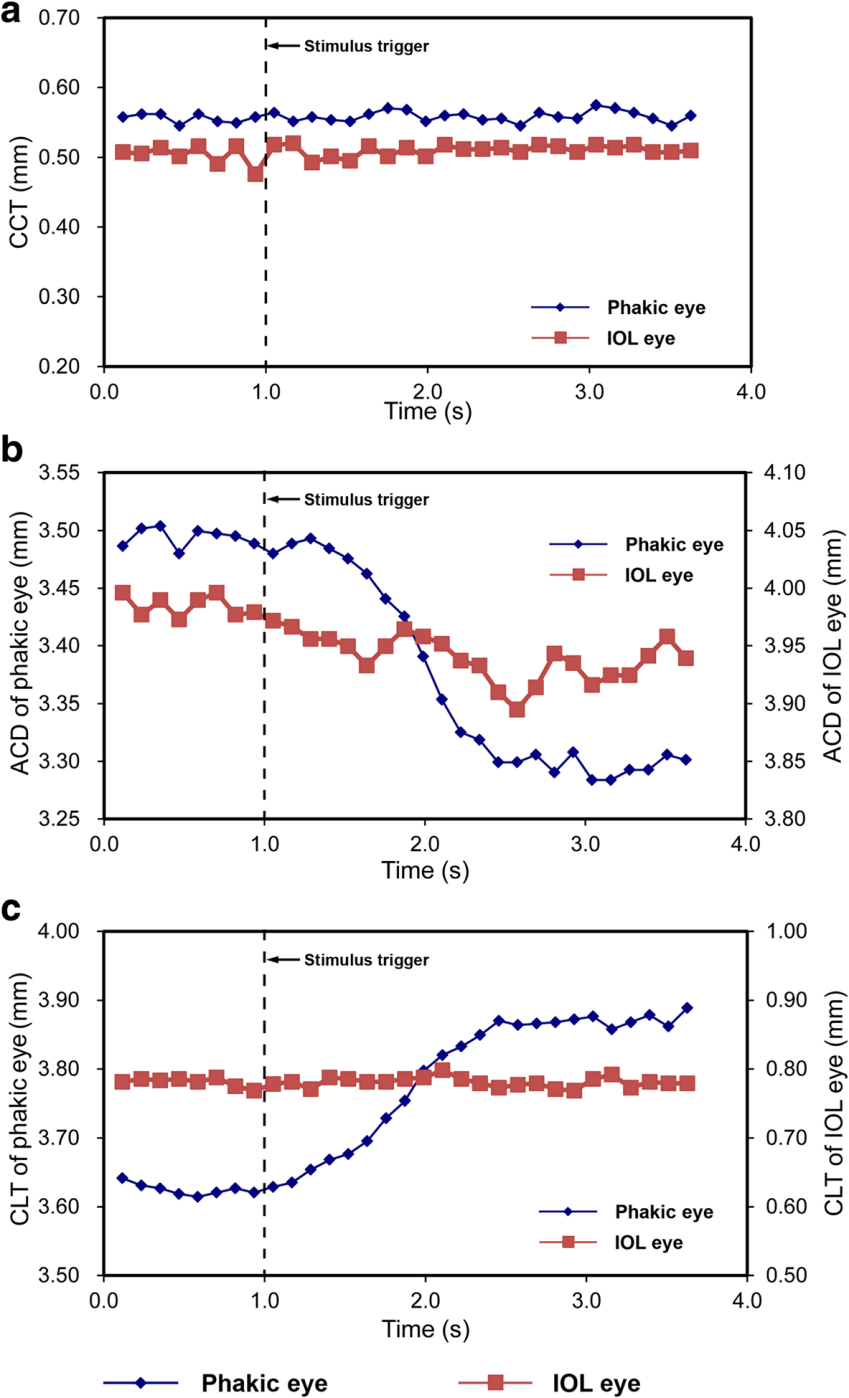
The dynamic changes of the axial biometry of the anterior segment depicted for both a phakic eye and an IOL implanted eye. **a**: the dynamic changes in central corneal thickness; **b**: the dynamic changes in anterior chamber depth; **c**: the dynamic changes in central lens thickness. Blue line: phakic eye; Red line: IOL implanted eye. CCT, central corneal thickness; ACD, anterior chamber depth; CLT, central lens thickness

As showed in Fig. 7, the IOL was clearly presented with overlapping images. Figure 8 showed the dynamic changes in the anterior segment of the phakic eye and the IOL implanted eye. The thickness of the cornea (Fig. 8a) did not change during accommodation. The decreased ACD (Fig. 8b, blue line) and increased CLT (Fig. 8c, blue line) were consistent with the sigmoidal function in the phakic eye. The ACD in the IOL implanted eye trended to decrease although the change was much smaller than that in the phakic eye (Fig. 8b, red line). The thickness of IOL remained unchanged during accommodation (Fig. 8c, red line).

## Discussion

The SD-OCT provided high data acquisition speeds and high axial resolutions. However, the limitation in scan depth affected the imaging of the entire anterior segment. Removing the complex conjugate artifacts in SD-OCT permitted the acquisition of deeper imaging depths, using high-speed CMOS cameras to catch multiple images and to eliminate complex ambiguities [10, 14, 17, 19, 23]. However, when a single OCT channel was used, the technique reduced the speed of the image. This approach achieved an axial scan depth up to approximately 10 mm but did not image the accommodation in some highly myopic eyes. Previously, we developed a dual-channel dual-focus OCT for imaging accommodation [13]. The reflected light in the sample arm was attenuated by 50% for each channel, which decreased the signal-to-noise ratio [13, 23]. Additionally, the two-channel system imaged the posterior lens region and the region from the cornea to the anterior lens but failed to image the central crystalline lens area due to a gap between the two simultaneous OCT images. The high-speed reflective Fabry-Perot tunable lasers allowed the optical frequency domain imaging system (also called swept source OCT) to attain longer image depths of 12 mm, but the axial resolution (9–14 μm) was worse than in the SD-OCT [15, 17, 21, 22, 36]. In the previous study, we tested a spectrometer with a 12 mm scan depth that imaged the entire ocular anterior segment. The system demonstrated good repeatability for measuring the anterior segment and was an excellent tool for studying accommodation [25].

Sensitivity is an important aspect of the SD-OCT, which determines the contrast of the image and the maximum detected depth. The intensity of light reflected back from deeper tissue was extremely low because the biological tissue was not completely transparent. The signal intensity decreased as the imaged depth increased, indicating that the signal-to-noise ratio decreased as the position moved farther away from the zero-delay line [18, 37]. By altering the placement of the mirrors at the reference arm, the axial plane imaging range could be extended by stitching the two images together [16, 20, 25, 27]. Cropping the images for stitching, as demonstrated previously, may result in a sensitivity valley at the center of the image [16]. If the scan depth is long enough, image overlap may be beneficial for normalizing the SNR and for future image registration, as demonstrated previously [20, 25] and in the present study. Based on this approach, the automatic software, which was recently developed, could extract and trace the contour of the iris and the lens anterior surface for further image transformation (including rotation and translation) between the two images and then image overlapping.

Low resolution was a drawback of the original system, which was overcome using cameras with more camera pixels and a wider bandwidth projecting on the camera line. The theoretical axial resolution of SD-OCT increases at wider bandwidths and lower central wavelengths [38]. In the present study, the SLD had a central wavelength of 840 nm and a bandwidth of 50 nm; the axial resolution of the light source was theoretically calculated to be 6.3 μm. However, the spectral range of the line array camera limited the use of the available bandwidth of the SLD because the truncated spectrum had a configuration similar to that of the spectrometer. The measured axial resolution was worse than the theoretical value for a CCD with 2048 pixels. This phenomenon where there is a decreased resolution due to less active camera pixels has been described elsewhere [10, 39]. In the present study, the axial resolution of the two systems using 4096 pixels array cameras was similar, which was close to the theoretical values that resulted in the almost full projection of the bandwidth of the light source.

Image acquisition speed is another important factor in designing a long scan depth system for imaging accommodation. The acquisition time should be short in the OCT application because the accommodative process is highly dynamic. The CMOS camera with a high data transfer rate makes it possible to investigate the changing ocular anterior segment as a function of the response time during dynamic accommodation. Some researchers have determined that the accommodative response increases as a function of time and can be fitted to a sigmoidal curve [40, 41]. In the present study, the sigmoidal function of the time dependent changes in lens thickness and the anterior chamber depth were evident during accommodation. Interestingly, the anterior chamber depth in the IOL implanted eye decreased slightly in response to the accommodation stimulus, implying that the IOL experienced forward movement. The phenomenon has also been reported elsewhere; even the IOL was designed as a mono-focus [42, 43]. This finding indicates that the CMOS system, with its high speed, may be suitable for imaging the subtle changes of the accommodative biometry. On the other hand, as the most important component, the crystalline lens reshapes its surface in a complex form with tilting and/or decentration. Thus, three-dimensional scan patterns are required, which the OCT based on CMOS camera can perform [10]. In the present study, the light exposure time of the CMOS was set to 44 μs, indicating that an acquisition time for a single image of 0.12 s, is short enough to image the human eye in real time or in a three-dimensional pattern scan.

In the static accommodation, we tested the imaging of the entire segment using the three systems with a scan speed of 2.7 FPS (6000 A-scan per second) for the CCD systems and 8.3 FPS (17,500 A-scans per second) for the CMOS system. The integration times for all three systems needed to increase so that the scan speed could be decreased. This approach of increasing integration time (resulting in the reduction of the scan speed) has been used in many previous studies including ours [10, 25]. Our dynamic accommodation experiment demonstrated that the response of accommodation would be as quick as 0.5 s and the slow CCD system with 2.7 FPS may not be fast enough for capturing the start point of the accommodative response to the stimulus. Based on these experiments, we demonstrated the impact of the scan speed on the image quality and real-time data acquisition. We also demonstrated that the minimal integration time for the three systems for acquiring images with high quality in the static accommodation experiment. Taken together, the CMOS system would be recommended for imaging real-time accommodation, while all three systems can be used for imaging static accommodation.

## Conclusions

This study describes the impact of enhanced axial resolution, speed and SNR on long scan depth SD-OCT, which images the entire ocular anterior segment in vivo during accommodation. We demonstrate the improved performance of the OCT system by enhancing the axial resolution with 4096 pixels camera and the scan speed by using the CMOS camera. All of the OCT systems tested with the SNR enhancement approach yielded similar biometric results in the model eye and the human eye, indicating that they may be used for imaging the static accommodation. For imaging real-time accommodation, the CMOS system may be recommended. In the future, the application of the SD-OCT systems with long scan depth, high resolution and high scan speed will be improved by implementing automatic image registration, segmentation and a 3-dimensional reconstruction in clinical applications.

## Abbreviations

ACD: Anterior chamber depth
CCD: Charge Coupled Device
CCT: Central corneal thickness
CLT: Central lens thickness
CMOS: Complementary Metal-Oxide-Semiconductor Transistor
IOL: Intraocular lens
MRI: Magnetic resonance imaging
OCT: Optical coherence tomography
OD: Optical density
OPD: Optical path difference
PSF: Point spread function
SD-OCT: Spectral domain OCT
SLD: Superluminescent diode
SNR: Signal-to-noise ratios
UBM: Ultrasound biomicroscopy
